# The diagnosis and treatment introspection of the first imported case of atypical cerebral schistosomiasis in Guangzhou city

**DOI:** 10.1371/journal.pntd.0006171

**Published:** 2018-03-15

**Authors:** Yuehong Wei, Na Huang, Shouyi Chen, Dehao Chen, Xiaoning Li, Jianmin Xu, Zhicong Yang

**Affiliations:** 1 Guangzhou Center for Disease Control and Prevention, Guangzhou, Guangdong province, China; 2 Department of Environmental Health, Rollins School of Public Health, Emory University, Atlanta, Georgia, United States of America; George Washington University, UNITED STATES

## Overview

### Objective

A rare case of cerebral schistosomiasis is reported from Guangzhou, which is a non-endemic region.

### Case report

A 70-year-old female with a half-month history of schistosomiasis presented to a nonendemic region for treatment. Brain computed tomography (CT) and MRI revealed a lesion in the left occipital lobe; other clinical testing was not remarkable. The diagnosis was an intracranial occupying lesion in the left occipital lobe. After the removal of the brain lesion via cranial surgery, it was noticed that the woman came from an endemic region of schistosomiasis. The discovery of eggs in the brain lesion and a positive ELISA result led to a final diagnosis of cerebral schistosomiasis.

### Conclusion

Although studies on the treatment of cerebral schistosomiasis exist, few reports have focused on the diagnosis of this illness in nonendemic settings. In this case, the exposure history of the patient was not taken into account at the beginning of treatment. This experience indicates the need for physicians to consider disease epidemiology in relation to patient history before conducting clinical interventions.

## Introduction

Schistosomiasis is a zoonosis in tropical and subtropical developing countries, including areas of Southern China. This neglected tropical disease has infected 240 million people, and 700 million people are at risk around the world [1]. When the People’s Republic of China (PRC) was founded in 1949, approximately 10 million people in the country were infected with the disease. After interventions performed by officials, the number of cases was reduced to 2.5 million at the end of the 1970s [2]. The predominant etiological agents include *Schistosoma japonicum*, *S*. *haematobium*, and *S*. *mansoni* [3]. *S*. *japonicum* is the only *Schistosoma* that exists in China. Cerebral schistosomiasis is caused by the accidental ectopic movement of the parasite’s eggs into the brain. Owing to this characteristic, this kind of schistosomiasis is rare. Although clinical studies of this rare disease have been conducted, few studies have focused on the diagnostic experience of this illness in nonendemic regions.

## Case report

### Ethics statement

Written informed consent to publish this case study and any supporting images was acquired from the patient. This study was approved by the Ethics Committee of the Guangzhou Center for Disease Control and Prevention (GZCDC) using the Declaration of Helsinki (protocol number: GZDCD-ER[A]2016006).

### Case presentation

**Fig 1:**
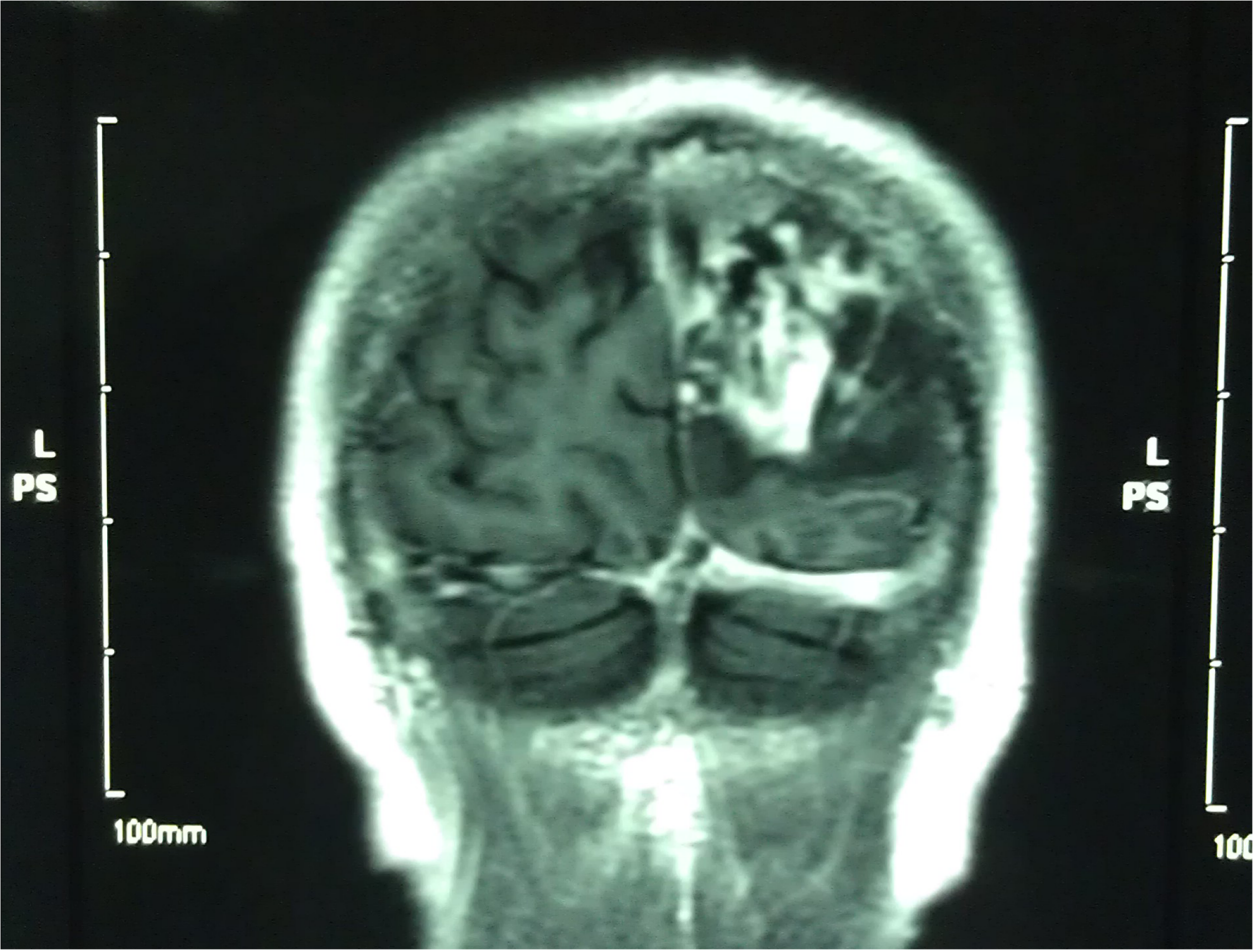
MRI of the brain before treatment. MRI of the brain before treatment shows an occupied lesion in the left occipital region.

A 70-year-old woman who lived in Jianli county, an endemic region for schistosomiasis in China, experienced a continuous headache at the top of the left occipital lobe beginning a half-month before presentation. She went to the county hospital for treatment, and the symptoms eased after the intervention. A week before presentation, the patient began to exhibit disorderly speech, her contralateral limbs became weak, and she became unable to see clearly out of her right eye, so she returned to the doctor. CT and MRI scans of her head showed an occupied lesion in the left occipital region of the brain (Fig 1), and she was hospitalized. In the clinical examination, her responses to the questions were only partly correct, and she was unable to calculate and memorize in a normal way. Inspection of the locomotor system showed below-normal muscle strength of the right limbs and normal strength in the left limbs. The other general physical examinations were unremarkable, including the absolute value of eosinophil (EO#), which can indicate parasite infection when it is higher than the normal upper range, which ranges from 0.03×10^9^/L to 0.2×10^9^/L (0.05×10^9^/L to 0.2×10^9^/L of the normal range). The patient had no history of epilepsy, infectious disease, or wounds due to surgery, and she lived in a schistosomiasis-endemic region. Cerebral MRI revealed a lesion in the top left of the occipital lobe with mottled nodule linear enhancement in the surrounding area. The primary diagnosis of this case before surgery was a brain lesion in the left occipital region of the brain.

Cranial microsurgery was performed to remove the brain lesion. The hard-textured nidus was yellow in color, with a size of 3 cm by 4 cm by 6 cm and obvious edema in the surrounding area, and its barrier was not distinctive from the surrounding tissue. The lesion was removed, and supportive care was provided. The patient’s condition was stable after the surgery. The incision recovered well, but the patient’s clinical symptoms did not improve significantly compared with her preoperation status. Inspection of the CT images showed low-density edema in the temporoparietal occipital lobe and a clear border with the surrounding tissue. Dehydration therapy was provided, but the edema peripheral to the nidus remained exacerbated. Because the patient was from a schistosomiasis-endemic region and the frozen section suggested chronic granulomatous inflammation, it was suspected that the illness was caused by cerebral schistosomiasis.

**Fig 2:**
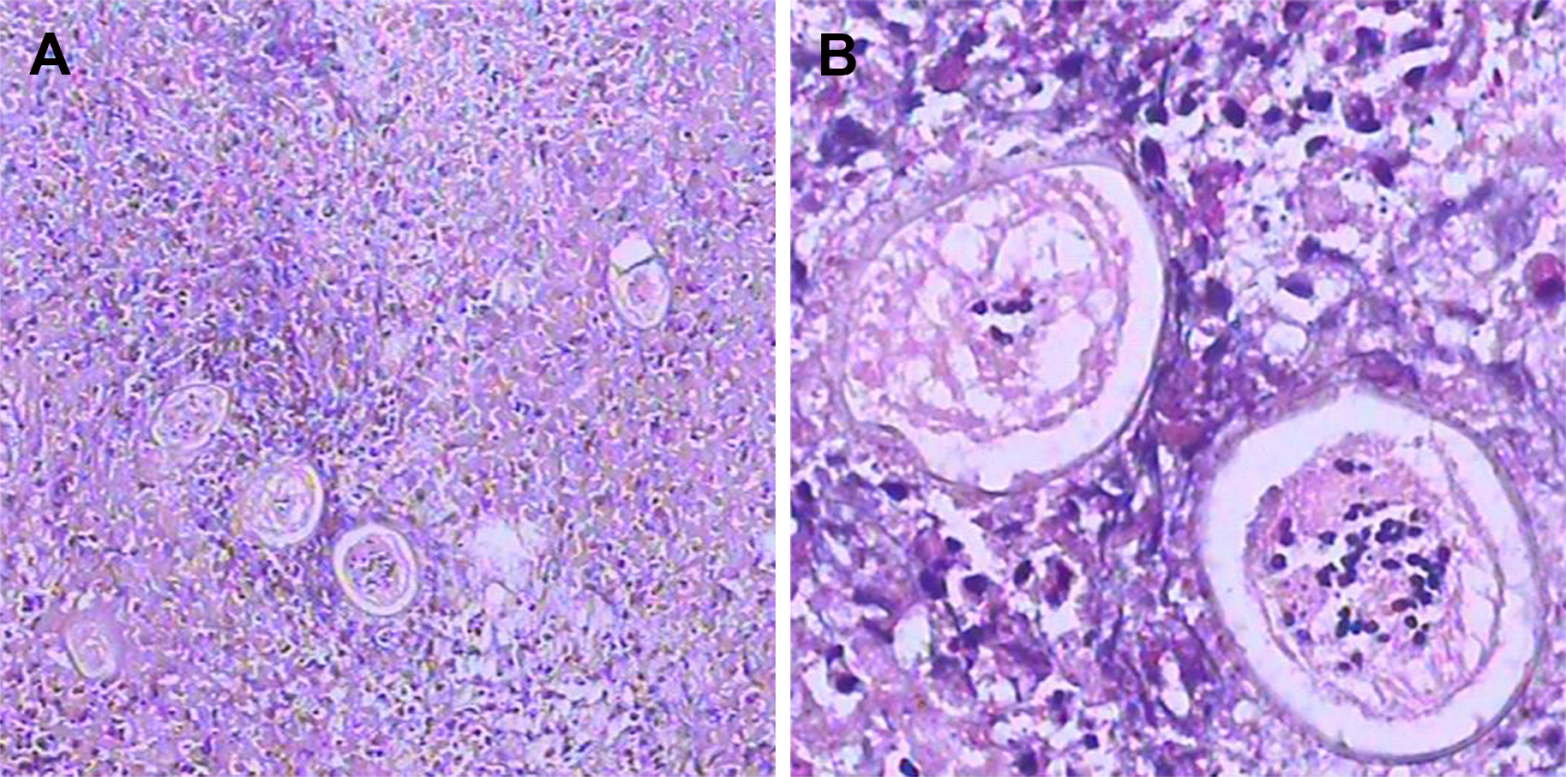
The paraffin section of formalin-fixed temporal lobe biopsy in low and high magnification view. (A) Low-magnification view of the paraffin portion of the formalin-fixed temporal lobe biopsy shows an immature *Schistosoma* egg surrounded by granuloma and glial cells. (B) High magnification shows two eggs with shells displaying the diagnostic acentric spine shape of immature *Schistosoma* eggs. Histiocytes near the eggs highlight that the nuclei of *Schistosoma* are smaller than those of the host’s cells.

**Fig 3:**
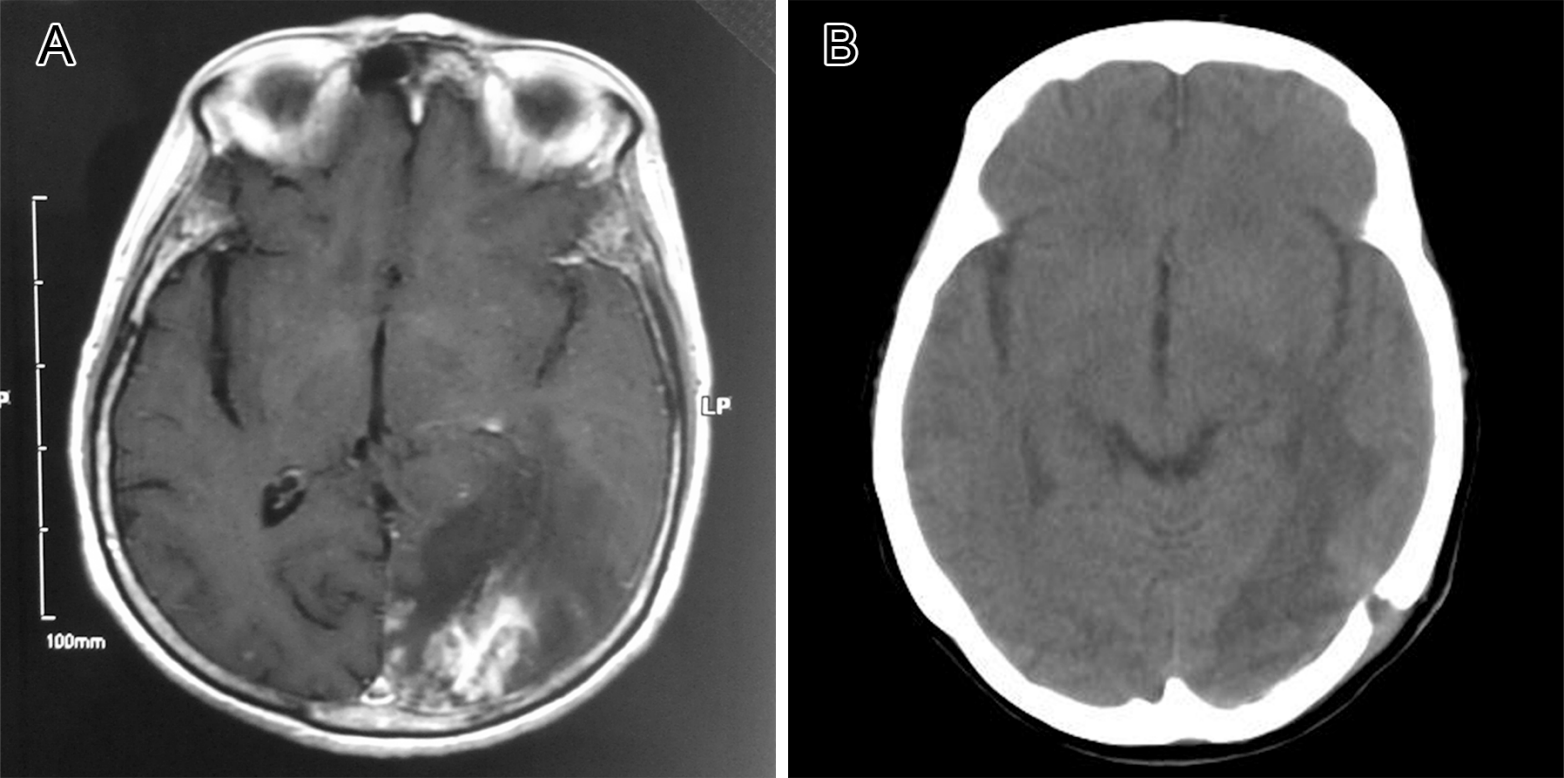
Comparison of brain imaging before and after treatment. (A) MRI before surgery showing an intracranial occupying lesion in the left occipital lobe, with edema and mottled nodular linear enhancement in the surrounding region. (B) CT result three months after surgery showing the disappearance of the nidus and fading of the edema. CT, computed tomography.

Praziquantel was administered for diagnostic treatment. Dehydration treatment was also reinforced, and it was observed that the patient’s condition was improving. ELISA testing was positive for antischistosomiasis antibodies, supporting the diagnosis of schistosomiasis. Furthermore, a review of several paraffin-embedded permanent sections showed refractive shells in the lesion and random unimpaired shells with the diagnostic acentric spine shape of immature *Schistosoma* eggs (Fig 2) [4]. Praziquantel was added with the dose of 7.7 g three times daily after the eggs were found, and other supportive treatments were continuously offered, since steroid therapy together with praziquantel can avoid complications and aid in faster patient recovery [5]. The patient’s condition improved: after three months, her verbal responses were on topic, and muscle strength in the right limbs was improved. The edema had alleviated compared with the previous inspection. The patient left the hospital after the clinical process was completed, and oral medication was continued. A CT performed with GE LightSpeed VCT-XT three months later showed that the nidus had disappeared, and the edema faded obviously compared with the previous MRI result (Fig 3). A three-year follow-up visit revealed that the physical condition of the patient was stable [6].

## Discussion

In normal situations, after the female parasite lays eggs in the small venues, they are moved to the intestine. When the eggs move to other parts of the body, ectopic manifestations of schistosomiasis occur. Cerebral schistosomiasis is rare and atypical among schistosomiasis cases and is caused by a host immunological response to the eggs after they cross the blood–brain barrier [7].

Due to the atypical nature of cerebral schistosomiasis, the diagnosis of this illness is difficult. In the present case, the doctor initially diagnosed the brain lesion as chronic granuloma before surgery; however, as a result of the pathology report obtained after the surgery, the doctor rediagnosed the lesion as cerebral schistosomiasis.

The exposure history was helpful in the diagnosis of this index case but was initially neglected. Schistosomiasis is mainly endemic in provinces crossed by the Yangtze River. In China, Jianli county, where the patient was from, is an endemic region for schistosomiasis (in 2013, the serum positive rate was 5.05%, and the estimated number of infections was 6,118) [8]. Hubei province, where Jianli county is located, has achieved control of transmission (the prevalence in humans and livestock is <1%, and the infection rate among residents is 0.17%) [9]. Nevertheless, in the present case, the main treatment was pursued in Guangzhou, Guangdong province, which is a nonendemic region of the parasite that has achieved transmission interruption (no local cases of schistosomiasis).

In the diagnosis of disease, it is important to fully understand the epidemiological exposure, especially for cases from areas outside the treatment location. In the present case, the disease was treated as a brain tumor before surgery; although information about *Schistosoma* exposure was gathered, it was neglected because the clinicians did not know the disease well.

**Key learning points**

- Nonendemic diseases should be considered when clinically diagnosing infectious diseases because of the increased movement of the population.
- Focusing on both exposure history and clinical detection can optimize the accuracy of diagnosis.
- In the present case, if the epidemiology of exposure had been considered from the beginning, the treatment scheme would have been optimized, and noninvasive treatment would have been considered before invasive brain surgery was performed.
